# In the Model Host *Caenorhabditis elegans*, Sphingosine-1-Phosphate-Mediated Signaling Increases Immunity toward Human Opportunistic Bacteria

**DOI:** 10.3390/ijms21217813

**Published:** 2020-10-22

**Authors:** Kiho Lee, Iliana Escobar, Yeeun Jang, Wooseong Kim, Frederick M. Ausubel, Eleftherios Mylonakis

**Affiliations:** 1 Division of Infectious Diseases, Rhode Island Hospital, Warren Alpert Medical School of Brown University, Providence, RI 02903, USA; kiho_lee@brown.edu (K.L.); iliana_escobar@brown.edu (I.E.); christine2372@gmail.com (Y.J.); 2 College of Pharmacy, Graduate School of Pharmaceutical Sciences, Ewha Womans University, Seoul 03760, Korea; wooseong_kim@g.ewha.ac.kr; 3 Department of Genetics, Harvard Medical School and Department of Molecular Biology, Massachusetts General Hospital, Boston, MA 02114, USA; ausubel@molbio.mgh.harvard.edu

**Keywords:** **Keywords**: Sphingosine-1-phosphate, S1P, S1P kinase, S1P transporters, pathogenic bacteria, immunity, *Caenorhabditis elegans*

## Abstract

Sphingosine-1-phophate (S1P) is a sphingolipid-derived signaling molecule that controls diverse cellular functions including cell growth, homeostasis, and stress responses. In a variety of metazoans, cytosolic S1P is transported into the extracellular space where it activates S1P receptors in a concentration-dependent manner. In the free-living nematode *Caenorhabditis elegans*, the *spin-2* gene, which encodes a S1P transporter, is activated during Gram-positive or Gram-negative bacterial infection of the intestine. However, the role during infection of *spin-2* and three additional genes in the *C. elegans* genome encoding other putative S1P transporters has not been elucidated. Here, we report an evolutionally conserved function for S1P and a non-canonical role for S1P transporters in the *C. elegans* immune response to bacterial pathogens. We found that mutations in the sphingosine kinase gene (*sphk-1*) or in the S1P transporter genes *spin-2* or *spin-3* decreased nematode survival after infection with *Pseudomonas aeruginosa* or *Enterococcus faecalis*. In contrast to *spin-2* and *spin-3*, mutating *spin-1* leads to an increase in resistance to *P. aeruginosa*. Consistent with these results, when wild-type *C. elegans* were supplemented with extracellular S1P, we found an increase in their lifespan when challenged with *P. aeruginosa* and *E. faecalis*. In comparison, *spin-2* and *spin-3* mutations suppressed the ability of S1P to rescue the worms from pathogen-mediated killing, whereas the *spin-1* mutation had no effect on the immune-enhancing activity of S1P. S1P demonstrated no antimicrobial activity toward *P. aeruginosa* and *Escherichia coli* and only minimal activity against *E. faecalis* MMH594 (40 µM). These data suggest that *spin-2* and *spin-3*, on the one hand, and *spin-1*, on the other hand, transport S1P across cellular membranes in opposite directions. Finally, the immune modulatory effect of S1P was diminished in *C. elegans* *sek-1* and *pmk-1* mutants, suggesting that the immunomodulatory effects of S1P are mediated by the p38 MAPK signaling pathway.

## 1. Introduction

Studies investigating the response of *Caenorhabditis elegans* to different pathogens, including bacteria and fungi, have revealed that conserved signaling pathways, such as the p38 mitogen-activated protein kinase (p38 MAPK) pathway and conserved transcription factors such as *hlh-30* (Helix Loop Helix-30, MITF/TFEB), play important roles in *C. elegans* immune responses [1,2]. In *C. elegans hlh-30* drives almost 80% of the host transcriptional immune response to a *Staphylococcus aureus* infection [1]. Furthermore, *hlh-30* mutant worms not only exhibit survival deficiencies against *S. aureus* but also Gram-negative bacteria, such as *Pseudomonas aeruginosa* [1]. Using microarray analysis, investigations have identified numerous other genes that are up-regulated or down-regulated during infection with a variety of bacterial species, including *Pseudomonas aeruginosa*, *Serratia marcescens*, *Staphylococcus aureus*, *Enterococcus faecalis*, and *Photorhabdus luminescens* as well as during infection by fungi such as *Candida albicans*, *Drechmeria coniospora*, and Harposporium spp. [2,3,4,5]. These studies have identified a variety of potential target genes that may play critical roles in the response to infection. Genes activated by both Gram-negative and Gram-positive bacteria are of interest for understanding general immune responses of the host to infection and may be potential therapeutic targets for new broad-spectrum antimicrobials.

An example of such a broadly activated immune-regulated *C. elegans* gene is SPINSTER-2 abbreviated as *spin-2* [3,5], which encodes a sphingosine-1-phosphate (S1P) transporter. S1P is synthesized by phosphorylation of the long chain base sphingosine by intracellular sphingosine kinase [6]. In mammals, intracellularly synthesized S1P is pumped from the inside of the cell out by S1P transporters, maintaining a higher level of S1P in the extracellular region than the inside of the cell [7]. In mammals, S1P is an important signaling molecule that is involved in a variety of cellular signaling processes including angiogenesis and anaphylaxis [8,9]. S1P transporters are highly conserved among many metazoans including nematodes and insects [10,11,12].

In *C. elegans*, there are four predicted S1P transporter homologs named *spin-1, -2, -3,* and *-4* [13,14]. Although there is extensive research on the role of S1P and related enzymes, to the best of our knowledge, there is yet to be any published work on the specificity and directionality of *spin-1-4* transporters. In *C. elegans*, research of S1P was initiated by looking at the expression and role of sphingosine kinase (*sphk-1*) and it was found that *sphk-1* is expressed in neuromuscular junction, muscles, neurons, hypodermal cells, the excretory canal, and intestinal cells [15]. Deficiency of *sphk-1* showed decreased lifespan caused by increased susceptibility to oxidative stress [16]. Notably, *sphk-1* promotes neurotransmitter release in neuromuscular junctions and activates the mitochondrial unfolded protein response (UPRmt) in the intestine [17,18,19].

In *C. elegans*, the UPRmt stabilizes cellular integrity by alleviating oxidative stress and by regulating metabolism during the immune response [20,21,22]. Therefore, we hypothesized that *sphk-1* and the 4 S1P transporters encoding genes *spin-1*, *-2*, *-3*, and *-4* may play an important role in the *C. elegans* immune response. Here, we demonstrate that deletion mutants of *sphk-1*, *spin-1*, *spin-2*, and *spin-3* exhibit aberrant immune responses in which exogenous S1P increases *C. elegans* resistance to *P. aeruginosa* and *E. faecalis*, and the immune modulatory activity of S1P is partially dependent on the p38 MAPK signaling pathway and the transcription factor *hlh-30*.

## 2. Results

### 2.1. Sphingosine Kinase and S1P Transporters Affect the C. elegans Immune Response

**Figure 1:**
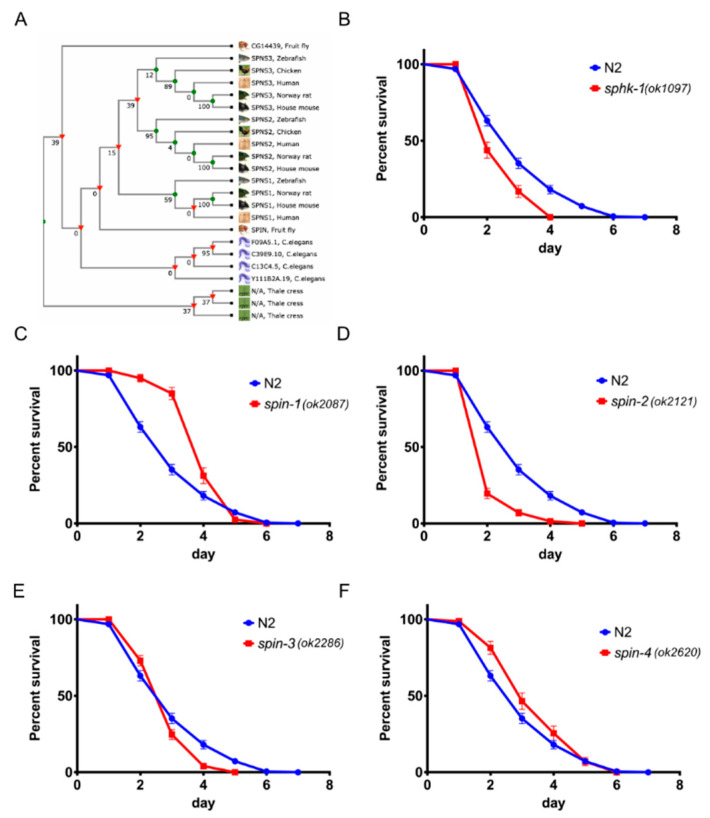
Sphingosine kinase and S1P transporters are related to the immune response in *C. elegans* on *P. aeruginosa* PA14. (**A**) Phylogenetic tree of S1P transporters. (**B**) Survival of worms with mutation in sphingosine kinase, *sphk-1*, and wild type N2. Survival of worms with mutation in S1P transporters *spin-1* (**C**), *spin-2* (**D**), *spin-3* (**E**), and *spin-4* (**F**) on *P. aeruginosa* PA14. The log-rank (Mantel-Cox) test was used for statistical analysis of worm survival.

In *C. elegans*, there are four putative S1P transporters, *spin-1*, *spin-2*, *spin-3*, and *spin-4*, which exhibit significant homology with other eukaryotic S1P transporters (Figure 1A). All four *C. elegans* S1P transporters exhibit >36.30% amino acid identity to human SPNS1 and SPNS2 (Supplementary Figure S1). Additionally, it was previously shown that *spin-2* is upregulated in *C. elegans* infected with *Serratia marcescens* [23] or *Enterococcus faecalis* [5].

To test whether the S1P signaling pathway is involved in *C. elegans* immunity, we exposed sphingosine kinase *sphk-1(ok1097)* mutant worms, which lack 90% of the sphingosine kinase coding region due to deletion of the start codon [17], to infection with the Gram-negative bacterium *P. aeruginosa* strain PA14. We found that *sphk-1* mutant animals were significantly more susceptible to *P. aeruginosa* PA14-mediated killing than wild type N2 worms (Figure 1B, *p <* 0.0001). In addition, we tested the role of S1P transporters during infection using deletion mutant animals made by the *C. elegans* Deletion Mutant Consortium [24]. We found that *spin-2(ok2121)* and *spin-3(ok2286)* mutant worms (*spin-2, p <* 0.0001 and *spin-3, p* = 0.0217) were also significantly more susceptible to *P. aeruginosa* PA14-mediated killing than wild type N2 worms. Unexpectedly, however, *spin-1(ok2087)* (*p <* 0.0001) and *spin-4(ok2620)* (*p* = 0.0062) mutant worms were more resistant to *P. aeruginosa* PA14 infection than the wild-type worms (Figure 1C–F).

**Figure 2:**
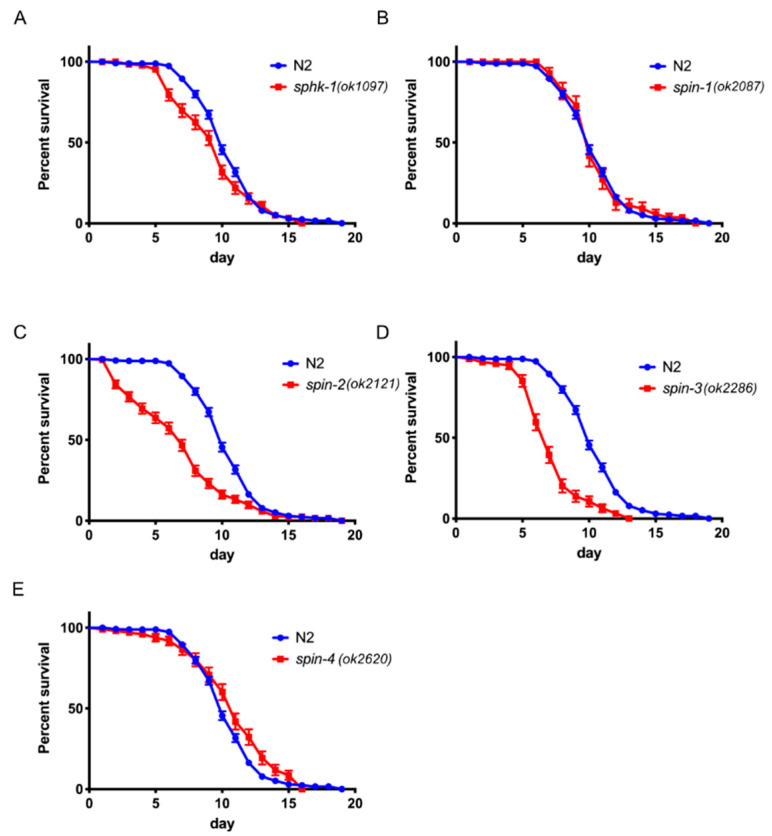
Sphingosine kinase and S1P transporters control the immune response toward *E. faecalis* MMH594 in *C. elegans*. Survival of worms with mutation in sphingosine kinase, *sphk-1* (**A**), or S1P transporters *spin-1* (**B**), *spin-2* (**C**), *spin-3* (**D**), and *spin-4* (**E**) and wild type N2 on *E. faecalis* MMH594. The log-rank (Mantel-Cox) test was used for statistical analysis of worm survival.

We also found that *sphk-1* (*p* = 0.0086), *spin-2* (*p* < 0.0001), and *spin-3* (*p* < 0.0001) mutant worms were significantly more susceptible to infection by *E. faecalis* strain MMH594, whereas *spin-4* (*p* = 0.0222) mutant worms were modestly more resistance than N2 (Figure 2A,C–E). However, unlike infection with *P. aeruginosa* PA14, *spin-1* (*p* = 0.8920) mutant worms did not show a difference in survival from wild-type (Figure 2B). The infection results with *P. aeruginosa* and *E. faecalis* indicate that the S1P signaling pathway plays a significant role in the *C. elegans* immune response against both Gram-negative and Gram-positive bacteria.

### 2.2. External Supplementation of S1P Extends Worm Survival

**Figure 3:**
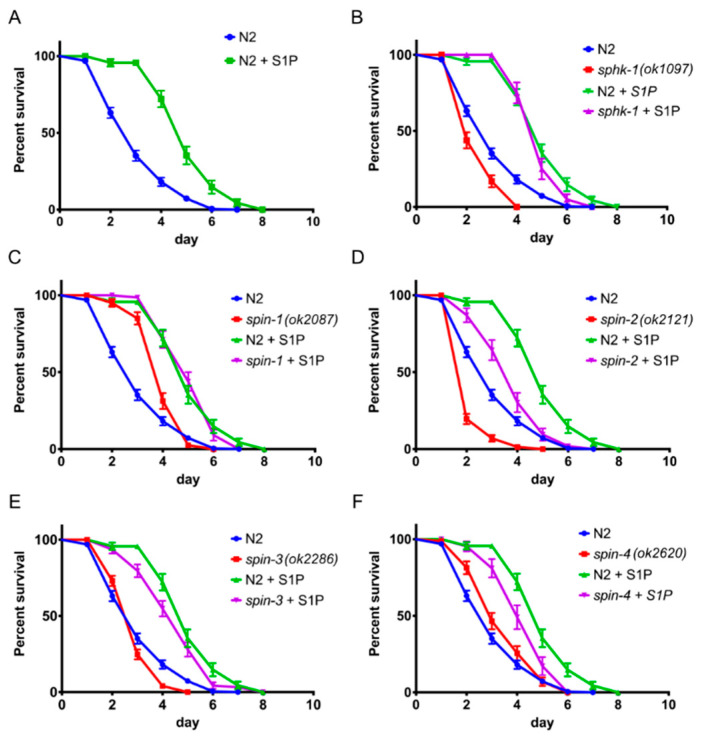
External supplementation of S1P stimulates the immune response to *P. aeruginosa* PA14 in *C. elegans.* The survival of worms with or without S1P supplementation in the background of wild type N2 worms (**A**), *sphk-1* (**B**), *spin-1* (**C**), *spin-2* (**D**), *spin-3* (**E**), and *spin-4* (**F**). The log-rank (Mantel-Cox) test was used for statistical analysis of worm survival.

The S1P kinase and S1P transporter mutant data in the previous section suggested that the level of S1P in or outside of intestinal epithelial cells, which is the primary site of the *C. elegans* immune response [18,19], may be critical for its activation. To test this hypothesis, we grew pathogenic bacteria *P. aeruginosa* and *E. faecalis* on various concentrations of externally supplemented S1P and control, solvent only, agar plates, which contained equal volumes of methanol without S1P. We found that, at a higher concentration such as 100 µM S1P, the methanol-only plates showed antimicrobial activity. For this reason, we chose to use 10 µM of S1P supplementation in the medium, which showed no antimicrobial effect in the methanol-only agar plate (data not shown). From our results, we found that a minimum of 10 µM of S1P supplementation dramatically extended wild-type worm survival by approximately two days (Figure 3A, *p <* 0.0001), suggesting that S1P is important for pathogen resistance.

**Figure 4:**
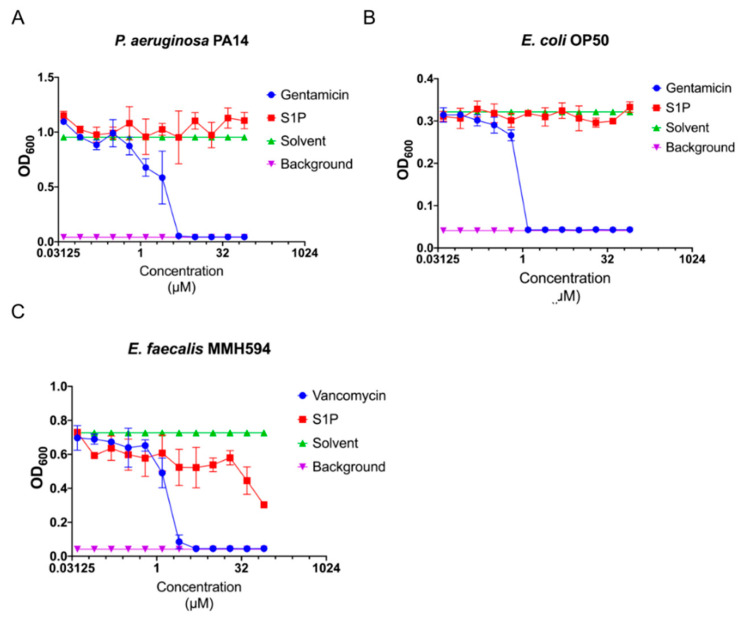
Spingoshine-1-phophate has limited antibiotic activity on *E. faecalis* MMH594 but not on Gram (–) bacteria. Growth of *P. aeruginosa* PA14 (**A**), OP50 (**B**), and MMH594 (**C**) exposed to antibiotics, gentamycin or vancomycin, solvent (methanol), and S1P at various concentrations after 18 h in Luria Broth or Brain Heart Infusion media. OD600, optical density at 600 nm.

We also tested the lifespans of *P. aeruginosa* PA14-infected *sphk-1* and *spin-1-4* mutant worms treated with S1P given that *sphk-1* mutants cannot produce any S1P and that *spin-1-4* mutant worms, presumptively, have impaired S1P transporter activity. We found that the lifespan of S1P treated *sphk-1* mutant worms was the same as wild-type worms treated with S1P (Figure 3B, *p_sphk-1 control_* vs. *_S1P_ <* 0.0001, *p_sphk-1_* vs. *_N2 with S1P_* = 0.2583), which is consistent with the conclusion that S1P fully rescues the hyper-susceptibility phenotype of the *sphk-1* mutant. On the other hand, mutation of *spin-2*, *spin-3*, and *spin-4* partially blocked the ability of S1P to rescue the infected worms (Figure 3D,E, *p_spin-2 control_* vs. *_S1P_ <* 0.0001, *p_spin-2_* vs. *_N2 with S1P_ <* 0.0001, *p_spin-3 control_* vs. *_S1P_ <* 0.0001, *p_spin-3_* vs. *_N2 with S1P_* = 0.0285, Figure 3F, *p_spin-4 control_* vs. *_S1P_* = 0.0012, *p_spin-4_* vs. *_N2 with S1P_* = 0.0010), whereas mutation of *spin-1* had no effect on the ability of S1P to enhance lifespan (Figure 4C, *p_spin-1 control_* vs. *_S1P_ <* 0.0001, *p_spin-1_* vs. *_N2 with S1P_* = 0.04272).

As explained in detail in the Discussion section, we think that the simplest interpretation of these data is that S1P transport into cells is important for activating the *C. elegans* immune response and that *spin-1* and *spin-4* encode canonical S1P cellular exporters. Mutation of *spin-1* or *spin-4* could, therefore, result in an accumulation of S1P inside of cells, leading to enhanced pathogen resistance, as shown in Figure 1C,F. In contrast, we hypothesize the *spin-2* and *spin-3* encode non-canonical S1P importers. A mutation of *spin-2* or *spin-3* could, therefore, results in the depletion of S1P levels in cells, leading to enhanced pathogen susceptibility, as shown in Figure 1D,E.

To further support the S1P supplementation results, we tested *sphk-1* and S1P transporter mutant worms infected with the Gram-positive *E. faecalis* MMH594. As anticipated, we observed that 10-µM S1P supplementation extended the lifespan of N2 worms when challenged with *E. faecalis* MMH594 (*p* < 0.0001), (Supplementary Figure S2A). *sphk-1* mutant worms, which showed reduced survivability when challenged with MMH594 (*p* = 0.0086), were fully rescued with 10 µM of exogenous S1P supplementation (*p* = 0.0536). The infection sensitive phenotype *spin-2* and *spin-3* mutant worms (*p* < 0.0001) were also fully rescued by the external supplementation of S1P, while the resistant phenotype of *spin-1* and *spin-4* mutant worms were insignificantly enhanced by extra S1P (Supplementary Figure S2C–F). Taken in their totality, these series of studies indicated that external S1P supplementation not only rescues deficiencies of the S1P mutant worms but also independently activated the immune response in *C. elegans*.

### 2.3. S1P Has No or Nominal Antimicrobial Activity

*C. elegans* pathogen resistance can be achieved by either a robust immune response or direct antimicrobial activity by a bioactive agent. In order to eliminate the possibility that S1P has direct antimicrobial activity against bacterial pathogens, we tested the antimicrobial activity of S1P by measuring a bacterial growth rate through optical scattering at 600-nm wavelength (OD_600_). Our results revealed no significant drop in absorbance when compared to the solvent, methanol (Figure 4A,B), when testing *P. aeruginosa* PA14 or *Escherichia coli* OP50 with S1P at various concentrations. When we examined *E. faecalis* MMH594, we found that S1P has antimicrobial activity at concentrations greater than 40 µM (Figure 4C, *p_S1P_* vs. *_vancomycin_* = 0.001011). However, S1P did not inhibit the growth of MMH594 at the 10 µM, which is the concentration that increases the *C. elegans* immune response to *E. faecalis* MMH594 (Supplementary Figure S2). These series of experiments imply that S1P exhibits no direct antimicrobial activity against the Gram-negative bacteria *P. aeruginosa* PA14 and *E. coli* OP50 and minimal activity against Gram-positive bacteria *E. faecalis* MMH595. This suggests that extracellular supplementation of S1P affects *C. elegans* pathogen resistance via an immune-modulatory mode of action rather than antimicrobial activity. To circumvent the issue of antimicrobial activity S1P toward *E. faecalis*, the following experiments were only carried out with the Gram-negative bacterium *P. aeruginosa* strain PA14.

### 2.4. S1P Signaling Depends on p38 MAPK Pathway and the Transcription Factor hlh-30

**Figure 5:**
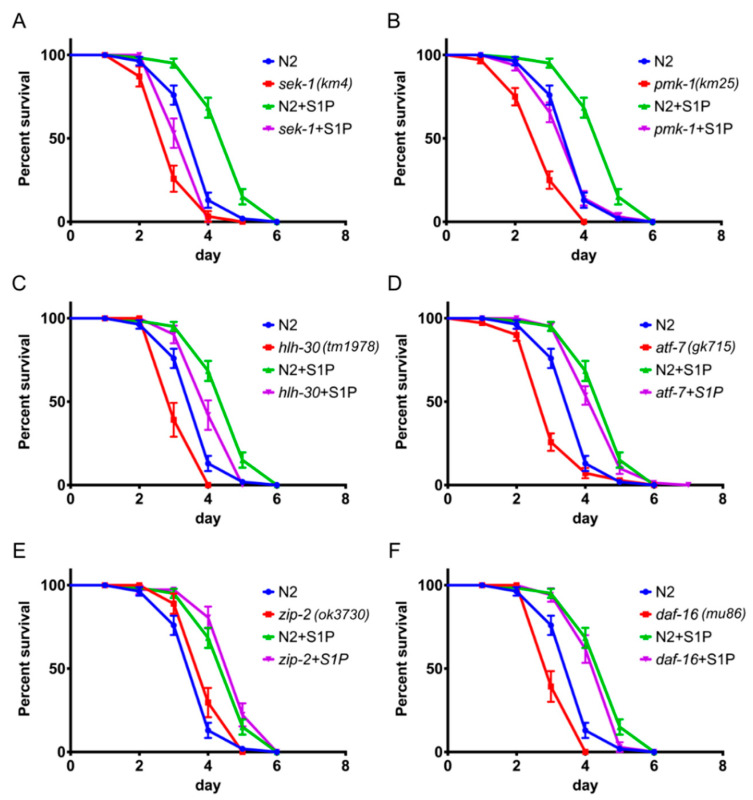
Increased immune response by S1P in *C. elegans* is dependent on p38 mitogen-activated protein kinase (MAPK) pathway and partially on *hlh-30*. The survival of worms with or without S1P supplementation in the background of wild type N2 and p38 MAPK pathway, *sek-1* and *pmk-1* (**A**, **B**), transcription factors *hlh-30* (**C**), *atf-7* (**D**), *zip-2* (**E**), and *daf-16* (**F**). The log-rank (Mantel-Cox) test was used for statistical analysis of worm survival.

Several immune signaling pathways have been identified in *C. elegans*, including the p38 MAPK and the insulin-like signaling pathways [25]. Key immune-related genes include *sek-1* and *pmk-1* (p38 MAPK) and the transcription factor *atf-7* (ATF2/CREB5) as well as *hlh-30* (MITF/TFEB), *zip-2* (bZIP), and *daf-16* (FOXO1/3/4) [25]. In order to examine whether S1P signaling is dependent on previously identified immunity-related signaling pathways, we challenged *C. elegans* strains with mutations in a variety of immune-related genes with *P. aeruginosa* PA14 during S1P supplementation. Mutations in all of these genes, except *zip-2*, cause a decrease in the survival rate in the absence of S1P (Figure 5). Significantly, *sek-1*, *pmk-1*, and *hlh-30* mutants exhibited decreased survival rate when challenged with *P. aeruginosa* PA14 and supplemented with S1P when compared to wild-type worms (*p <* 0.0001, *p* < 0.0001, and *p* = 0.0017, respectively, Figure 5A–C). On the contrary, the *P. aeruginosa* PA14-susceptible phenotypes of *atf*-7 and *daf-16* mutants were fully rescued by S1P supplementation on *P. aeruginosa* PA14 to the same levels of N2 worms supplemented with S1P (Figure 5D,F). Furthermore, *zip-2* mutant worms exhibited no significant difference in the survival rate when challenged with *P. aeruginosa* PA14 (*p = 0.*0738) and supplemented with S1P compared to wild-type worms (*p* = 0.1786). These results suggest that, in *C. elegans*, the S1P-mediated immune response is partially dependent on the p38 MAPK pathway and the transcription factor *hlh-30*.

### 2.5. S1P Signaling Affects C. Elegans Immune Effect Gene Lys-2

**Figure 6:**
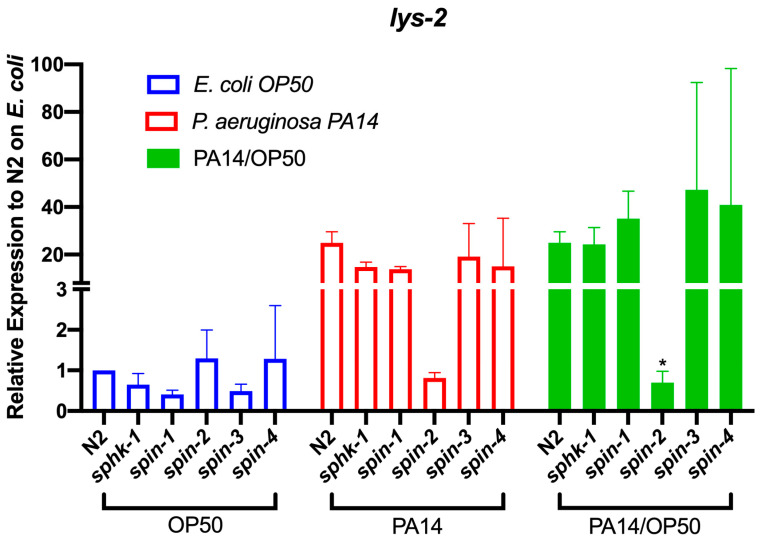
Transcriptional activation of immune response genes, *lys-2*, was diminished in the *spin-2* mutant worm. The fold changes of *lys-2* in wild type, sphingosine kinase (*sphk-1*) and S1P transporters mutant (*spin-1-4*) worms were normalized to house-keeping gene, *pmp-3*, and Y45F10D.4. The unpaired t-test was used for statistical analysis.

To investigate the transcriptional expression changes of p38 MAPK-dependent immune response genes by S1P signaling, we measured and compared the mRNA levels of seven immune response genes in *sphk-1* and S1P transporters (*spin-1-4*) mutant animals in response to exposure to *E. coli* OP50 or *P. aeruginosa* PA14 for 24 h. Among the genes tested, *lys-2*, a lysozyme encoding gene, was transcriptionally activated by the exposure to *P. aeruginosa* PA14 compared to *E. coli* OP50 in wild type N2 worms (Figure 6). In the *spin-2* mutant animals, the activation of *lys-2* after exposure to *P. aeruginosa* PA14 was inhibited (*p = 0.0183*). Other immune response genes tested were not significantly changed in the *spin-2* mutant nor did we find variable expression levels in either the *spin-1*, *3*, *4* and *sphk-1* mutants among multiple genes (Supplementary Figure S3).

### 2.6. S1P Signaling Has a Modest Effect on C. Elegans Lifespan in a Normal Food Source

**Figure 7:**
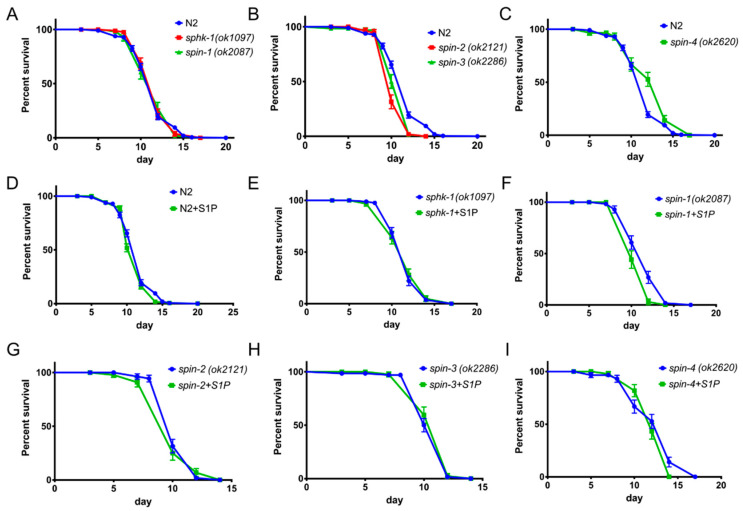
Select S1P transporters affect *C. elegans* lifespan while S1P supplementation has no affect. Mutant worms showing the same lifespan (**A**), decreased lifespan (**B**), and increased lifespan (**C**) compared to wild type N2. Comparison of the survival with or without S1P in the background of wild type N2 (**D**), *sphk-1* (**E**), *spin-1* (**F**), *spin-2* (**G**), *spin-3* (**H**), and *spin-4* (**I**). The log-rank (Mantel-Cox) test was used for statistical analysis of worm survival.

In *C. elegans*, the unfolded protein response through the p38 MAPK pathways connects aging and immunity [26]. Since external supplementation of S1P enhanced longevity during *P. aeruginosa* and *E. faecalis* infections, we tested whether this increase in lifespan was a consequence of a specific effect on the immune response or a general effect of overall increased lifespan. In these series of experiments, we fed worms with heat-killed *E. coli* OP50 supplemented with S1P in order to exclude any pathogenic traits of live *E. coli*. First, we found that, in the absence of S1P, *C. elegans sphk-1* and *spin-1* mutants exhibited similar lifespans to the wild type N2 strain (Figure 7A, *p_sphk-1_* = 0.4757, *p_spin-1_* = 0.8768), whereas *spin-2* and *spin-3* exhibited modestly shorter life spans than wild-type (Figure 7B, *p_spin-2_ <* 0.0008, *p_spin-3_* = 0.0054) and *spin-4* lived modestly longer (Figure 7C, *p_spin-4_* = 0.0005). Notably, however, external supplementation with S1P did not increase the lifespan of wild-type, *sphk-1*, *spin-2*, *spin-3*, or *spin-4* worms (Figure 7D,E,G,H,I) and modestly decreased the lifespan of the *spin-1* mutant (Figure 6F, *p = 0.0191*). These results indicate that the effects of S1P as well as the *sphk-1* and *spin-1-4* mutants on lifespan during the course of an infection are due to immuno-modulatory effects rather than a non-specific effect on worm longevity.

## 3. Discussion

The elucidation of the role of S1P signaling in immune responses in mammals led to the development of immunosuppressive agents such as Fingolimod (FTY720) [27] as well as highlighting potential novel therapies for intestinal *Salmonella* infections [28]. Here, we show that sphingosine kinase and S1P transporters are important components of the *C. elegans* immune response. Exogenous S1P supplementation not only fully compensated the immune deficiency observed in sphingosine kinase (*sphk-1)* mutants, but also dramatically increased the resistance of wild type worms to pathogenic bacteria. These results indicate that the S1P signaling pathway is involved in the *C. elegans* immune response to bacterial infections.

In mammals, S1P activates the immune response through extracellular G protein coupled S1P receptors [7] that regulate many biological processes including cell growth, apoptosis, stress responses, cell migration, and development [7]. There are more than 2000 G protein coupled receptors (GPCRs) in the genome of *C. elegans* [29], whereas only approximately 750 GPCRs have been identified in humans [30]. However, thus far, a S1P receptor has not been identified in *C. elegans* [31]. The work reported in this paper showed that external supplementation of S1P is sufficient to activate *C. elegans* immunity. In addition to S1P transport, it is possible that S1P is directly activating *C. elegans* immunity through an uncharacterized GPCR. Identification of S1P receptor(s) in *C. elegans* will give more clues in understanding how S1P signaling is related to the immune response.

In *C. elegans*, during a stress response, increased S1P in the cytosol of intestinal epithelial cells activates genes related to the intestinal UPR^mt^ [19]. Kim et al. showed that *sphk-1* localizes to the outer membrane of mitochondria [18] and subsequent production of S1P is important for the activation of intestinal UPR^mt^ [19]. In addition, they found that this phenotype is specific to intestinal cells and not neuronal cells [19]. Their working model suggests that S1P is both produced and is active inside intestinal epithelial cells in support of our model that intracellular S1P activates *C. elegans* immunity. One UPR^mt^ gene, in particular HSP-60, physically binds to SEK-1 and activates PMK-1 during the immune response [32]. Therefore, it is possible that increased levels of S1P within the cytosol by external supplementation activates the p38 MAPK pathway through the UPR^mt^.

Furthermore, we showed that the increased resistance elicited by S1P supplementation is at least partially dependent on the p38 MAPK signaling genes *sek-1* and *pmk-1* (Figure 5). In addition, we found that the transcriptional expression of a defense response encoding protein, LYS-2, depends on *spin-2*. *lys-2* is a marker of immune response, which is increased during *P. aeruginosa* infection [33] in a *pmk-1*-dependent manner [2]. Given previous studies by Kim et al. and our own findings, we speculate that the enhanced immune response elicited by exogenous supplementation of S1P might be related to p38 MAPK-dependent gene activation. Additional genomics studies are needed to further confirm this hypothesis.

**Figure 8:**
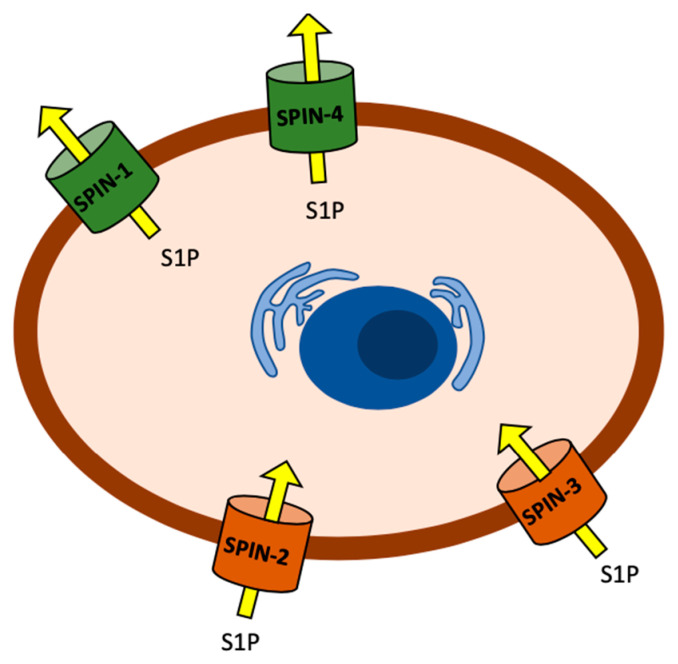
Model of S1P transporters directionality in *C. elegans.* SPIN-1 and SPIN-4 show canonical directionality transporting S1P out into the extracellular space. Mutations in these transports increases cytosolic S1P. SPIN-2 and SPIN-3 pump S1P into the cytosolic space. Mutations in both these transports decreases cytosolic S1P.

Our results show that the pathogen susceptible phenotypes of *sphk-1*, *spin-2*, and *spin-3* mutants are similar, whereas *spin-1* and *spin-4* exhibit a resistant phenotype when fed *P. aeruginosa* (Figure 1). Since *sphk-1* mutant worms are unable to produce S1P, the level of S1P inside of the cells should be low or absent. One model (Figure 8) that is consistent with these results is *spin-1* and *spin-4* encode canonical S1P transporters that export S1P out of the cytosol, whereas *spin-2* and *spin-3* encode non-canonical S1P transporters that import extracellular S1P. In *spin-2* and *spin-3* mutant worms, extracellular S1P transport into the cytosol may be decreased enough that *C. elegans* are unable to activate and generate resistance to pathogens. In contrast, the resistant phenotype of *spin-1* and *spin-4* may be due to the accumulation of S1P inside the cell.

If *spin-2* and *spin-3* are S1P importers, we would also expect that mutating them could decrease intracellular S1P accumulation in the presence of exogenous S1P, and, therefore, partially blocking the effect of the exogenous S1P, as shown in Figure 3. The fact that the *spin-1* mutation did not affect the ability of S1P to enhance the worm lifespan in the presence of exogenous S1P is also consistent with *spin-1* encoding a canonical S1P exporter.

The model shown in Figure 8 assumes that S1P is active in the cytosol and not extracellularly during the immune response in accordance with data published by Kim et al. and discussed above [19]. Our data are not consistent with models in which extracellular accumulation of S1P is sufficient to activate *C. elegans* immunity. For example, if extracellular S1P activates *C. elegans* immunity, we would predict that *spin-2* and *spin-3* mutants supplemented with extracellular S1P would show an increased immune response after *P. aeruginosa* infection, which is similar to wild-type worms under similar conditions. Conversely, the model in Figure 8 predicts that mutations in the S1P exporters *spin-1* and *spin-4* would also exhibit decreased survival. However, we found an extended lifespan after *P. aeruginosa* infection in *spin-1* and *spin-4* mutant worms, suggesting that extracellular S1P is not fully responsible for *C. elegans* immune activation and subsequent pathogen resistance.

The model in Figure 8 does not directly address the particular cell types in which S1P is functioning, but it is reasonable to assume that the intestinal epithelial cells are the most likely candidate since the *C. elegans* immune response to *P. aeruginosa* appears to be primarily localized to these cells based on previous reports [19] and our own results. The model also does not address in which tissues or cells S1P is being synthesized in and whether these cells are the same as those in which S1P exerts its immune-enhancing effects. An additional limitation is the impossibility to physically test intra-cellular or extra-cellular S1P levels in vivo or in vitro in *C. elegans*, which could possibly further support our model [16,34]. One possibility consistent with the model in Figure 8 is that S1P is synthesized in intestinal epithelial cells as intrinsic signaling molecules to enhance immunity. It is important to point out the other models that are consistent with our data, including models in which S1P functions intracellularly and the *spin-1/spin-4* transporters and *spin-2/spin-3* transporters, on the other hand, have the opposite polarities, as shown in Figure 8. However, in models in which the polarities of the transporters are flipped, it is also necessary to postulate that S1P functions extracellularly, which seems unlikely [19].

In any case, our data strongly suggests that SPIN-2/SPIN-3 and SPIN-1/SPIN-4 transport S1P in opposite directions. One potential discrepancy with this model, however, is the observation that *spin-4* mutants exhibit decreased longevity when compared to N2 in the presence of exogenous S1P when fed *P. aeruginosa*. In these experiments, *spin-4* exhibits the same phenotype as *spin-2* and *spin-3* mutants. However, *spin-4* mutants showed more increased longevity than N2 with external supplementation of S1P when fed with *E. faecalis* (Supplementary Figure S2F). We speculate that the different lifespans of *C. elegans spin-4* mutants to different pathogenic bacteria may be caused by the distinctive immune gene activation profiles between Gram-positive *E. faecalis* or Gram-negative *P.* aeruginosa in *C. elegans* [2,23,33,35,36,37]. Moreover, microarray data also suggest that even bacterial strains within the same category (Gram-negative or Gram-positive) can also elicit different immune gene activation in *C. elegans* [36,38].

It has been previously observed that *sphk-1* strain has a decreased lifespan on live *E. coli* HB101 [16]. However, we found that *sphk-1* mutant animals (as well as the *spin-1-4* mutant animals) had the same lifespan as wild-type worms when fed heat-killed *E. coli* OP50. Since a live *E. coli* food source is pathogenic as worms mature (>9 days) [39,40], it is possible that the decreased lifespan of *sphk-1* on live *E. coli* HB101 is a consequence of *E. coli-*mediated killing.

In conclusion, although direct evidence is still needed to support the relationship between intracellular concentrations of S1P and pathogenic infection, our findings provide novel insights into the studies of sphingosine signaling and the response to infection. In addition to the immune response, understanding the molecular basis of S1P signaling in *C. elegans* may yield novel insights into conserved features of the innate immune response.

## 4. Materials and Methods

### 4.1. Nematode Strains

The following *C. elegans* strains were used in this study. N2 wild-type, VC916 *sphk-1(ok1097)*, RB1678 *spin-1(ok2087)*, RB1702 *spin-2(ok2121)*, RB1778 *spin-3(ok2286)*, RB1986 *spin-4*(ok2620), KU25 *pmk-1(km25)*, KU4 *sek-1(km4)*, JIN1375 *hlh-30(tm1978)*, VC1518 *atf-7(gk715)*, VC3056 *zip-2(ok3730)*, and CF1038 *daf-16(mu86)* were obtained from *C. elegans* Genome Center (CGC).

### 4.2. Bacterial Strains and Maintenance

*Pseudomonas aeruginosa* PA14 and *Escherichia coli* OP50 were cultured in Luria Broth (LB) with shaking at 37 °C for 16 to 20 h. *Enterococcus faecalis* MMH594 was incubated in Brain Heart Infusion (BHI) media with shaking at 37 °C for 16 to 20 h. The overnight culture was measured by optical density in a 600-nm wavelength (OD_600_) and adjusted to OD_600_ = 1.5 to control the number of bacteria added to each plate. Adjusted inoculum was then spread on Nematode Growth Media (NGM) or Slow Killing (SK) media agar plates that contain 50 µg/mL of 5-Fluoro-2’-deoxyuridine (FUDR, Sigma Aldrich #F0503, Saint Louis, USA). SK plates contained 0.35% of peptone while NGM plates contained 0.25% [32]. Fresh bacterial plates were regularly streaked on a weekly basis from –80 °C glycerol stocks on solid agar containing nutrient broth. Plates were stored at 4 °C.

### 4.3. Reagents and Media Preparation

Sphingosine-1-phosphate (Sigma Aldrich #73914, Saint Louis, MO USA) was dissolved in methanol due to S1P’s poor solubility in water to a final stock concentration of 2 mM. S1P was diluted into solid agar NGM or SK after autoclaved medium was cooled down to 45 °C in order to prevent degradation of S1P. S1P-contatining NGM or SK was poured onto assay plates using a serological pipette. The S1P stock solution in methanol was directly diluted into liquid LB, or BHI to desired concentrations for minimum inhibitory concentration (MIC) tests. An equivalent percentage of methanol was used as a vehicle control in all experiments.

### 4.4. C. elegans Lifespan and Killing Assay

Methods used were as previously described in Lee et al. 2017 [41]. Synchronized L1 *C. elegans* were grown on NGM plates containing *E. coli* OP50. After 24 h of incubation at 25 °C, worms were treated with FUDR to block self-fertilization. After an additional 24 h incubation at 25 °C, worms reached a young adult stage and were then added to assay plates. *P. aeruginosa* PA14 assay plates were prepared by adding 100 µL of adjusted *P. aeruginosa* PA14 inoculum onto FUDR (50µg/mL) containing SK plate and spread to the edge. Plates were incubated at 37 °C for 24 h, and then incubated an additional 16 h at 25 °C to maximize virulence of *P. aeruginosa* PA14. To prepare assay plates with *E. faecalis* MMH594, 100 µL of *E. faecalis* MMH594 inoculum was added onto FUDR (50 µM) containing BHI plates. Plates were incubated at 37 °C for 24 h and then cooled to room temperature before the addition of worms. More than 50 young adult worms were added onto plates containing either heat-killed *E. coli* OP50 for the lifespan assay or pathogen *P. aeruginosa* PA14 or *E. faecalis* MMH594 for the killing assay and incubated at 25 °C. Dead worms were counted daily. Worms that presented no response to physical stimuli after gentile prodding on the anterior regions of *C. elegans* with a platinum wire were scored as dead. The log-rank (Mantel-Cox) test was used for statistical analysis of worm survival. These experiments were not conducted in a blind fashion.

### 4.5. Measuring Minimum Inhibitory Concentration

Minimum inhibitory concentrations were determined by the standard micro-dilution method published by the Clinical and Laboratory Standards Institute [42]. Each assay was conducted in triplicate. These experiments were not conducted in a blind fashion.

### 4.6. Sequence Alignment

All sequence alignments and evaluations were done using open access software Clustal Omega (http://www.clustal.org/omega/) [43].

### 4.7. RNA Preparation, cDNA Synthesis, and Quantitative PCR

Young adult worms treated for 24 h with *E. coli* OP50 or *P. aeruginosa* PA14, as done in the *C. elegans* Killing Assay protocol, were collected and washed with phosphate buffer saline (PBS). Supernatant was aspirated leaving approximately 50 µL and making sure not to disturb the *C. elegans* pellet. A total of 500 µL of Trizol (Invitrogen, Carlsbad, CA USA) was then added to each sample. Samples were frozen at −80 °C and then immediately thawed at 37 °C. This freeze-thaw cycle was repeated one additional time for a total of two freeze-thaw cycles. Next, 150 µL of additional Trizol was added to each sample and allowed to incubate for 5 min at room temperature. 140 µL of chloroform was then added to each sample, vortexed vigorously for 15 s, and then incubated for 2 min at room temperature. Samples were then centrifuged at 11,000 rpm for 15 min at 4 °C. Clear supernatant was collected into clean microtubes and 70% ethanol was added at a 1:1 ratio. Samples were mixed and run through RNeasy spin columns (Qiagen # 74104, Germantown, MD, USA) and washed following manufacturer’s instructions. RNA concentration and 260/280 ratios were assessed to determine RNA quality. cDNA was prepared using Verso cDNA synthesis Kit (Thermo Fisher, Waltham, MA, USA). Equal concentrations of RNA were used to synthesis cDNA. Real-time PCR was performed using iTaq Universal Syber Green Supermix (BioRad, Hercules, CA, USA) and CFX96 Real-time PCR machine following manufacturer’s instructions. Primers used for real-time PCR can be found in Table 1.

## Figures and Tables

**Table 1:** qPCR primer list.

References	Sequence Name	Gene Name	Direction	Sequence
[44]	Y22F5A.5	*lys-2*	F	GCTGGATTGGGAATTGAGAC
	Y22F5A.5	*lys-2*	R	GACGTTGGCAGTTGGATTG
[23]	F35C5.6	*clec-63*	F	AGGAGCTGCTCTTCAAACCA
	F35C5.6	*clec-63*	R	TCCAGGATGAGGAGATGGTG
[45]	T07C4.4	*spp-1*	F	TGGACTATGCTGTTGCCGTT
	T07C4.4	*spp-1*	R	ACGCCTTGTCTGGAGAATCC
[46]	K08D8.5		F	CCGGGAAGTCGAATGAACAT
	K08D8.5		R	GATGCAACACCTGCCAAAGA
[47]	F08G5.6	*irg-4*	F	CACAATGATTTCAATGCGAGA
	F08G5.6	*irg-4*	R	GTTTCGACCGAGAAATCGAG
[47]	F55G11.2		F	TGGTTCTCCAGACGTGTTCA
	F55G11.2		R	CAGCCTTGCCTTTACTGACA
[48]	C54G10.3	*pmp-3*	F	GTTCCCGTGTTCATCACTCAT
	C54G10.3	*pmp-3*	R	ACACCGTCGAGAAGCTGTAGA
[48]	Y45F10D.4		F	GTCGCTTCAAATCAGTTCAGC
	Y45F10D.4		R	GTTCTTGTCAAGTGATCCGACA
[23]	F56D6.2	*clec-67*	F	CGGGCTGGGAATATATCAAT
	F56D6.2	*clec-67*	R	CAATAGGTTGTGGCGTATGG

## Supplementary Materials

Supplementary Materials can be found at https://www.mdpi.com/1422-0067/21/21/7813/s1. Figure S1. Amino acid alignment of *C. elegans* S1P transporters with human SNPS1 and SNPS2. Percent identity matrix included below alignment. Figure S2. S1P stimulates immune response to *E. faecalis* MMH594 in *C. elegans*. The survival of worms with or without S1P in the background of wild type N2 worms (**A**), *sphk-1* (**B**), *spin-1* (**C**), *spin-2* (**D**), *spin-3* (**E**), and *spin-4* (**F**). The log-rank (Mantel-Cox) test was used for statistical analysis of worm survival. Figure S3. Relative expression of immune response genes. The relative mRNA level immune response genes in wild type N2, *sphk-1*, *spin-1*, *spin-2*, *spin-3*, and *spin-4* was measured by quantitative PCR. The fold changes of each genes in wild type, sphingosine kinase (*sphk-1*), and S1P transporters mutant (*spin-1-4*) worms were normalized with house-keeping gene, *pmp-3*, and Y45F10D.4.

## Abbreviations

S1P	Sphingosine-1-phosphate
*C. elegans*	*Caenorhabditis elegans*
*P. aeruginosa*	*Pseudomonas aeruginosa*
*E. faecalis*	*Enterococcus faecalis*
UPR^mt^	Mitochondrial Unfolded Protein Response
