# Supplementary figures and images for: In the Model Host Caenorhabditis elegans, Sphingosine-1-Phosphate-Mediated Signaling Increases Immunity toward Human Opportunistic Bacteria
Source: Int J Mol Sci. 2020 Oct 22;21(21):7813. doi: 10.3390/ijms21217813 (PMC7672543; doi:10.3390/ijms21217813)

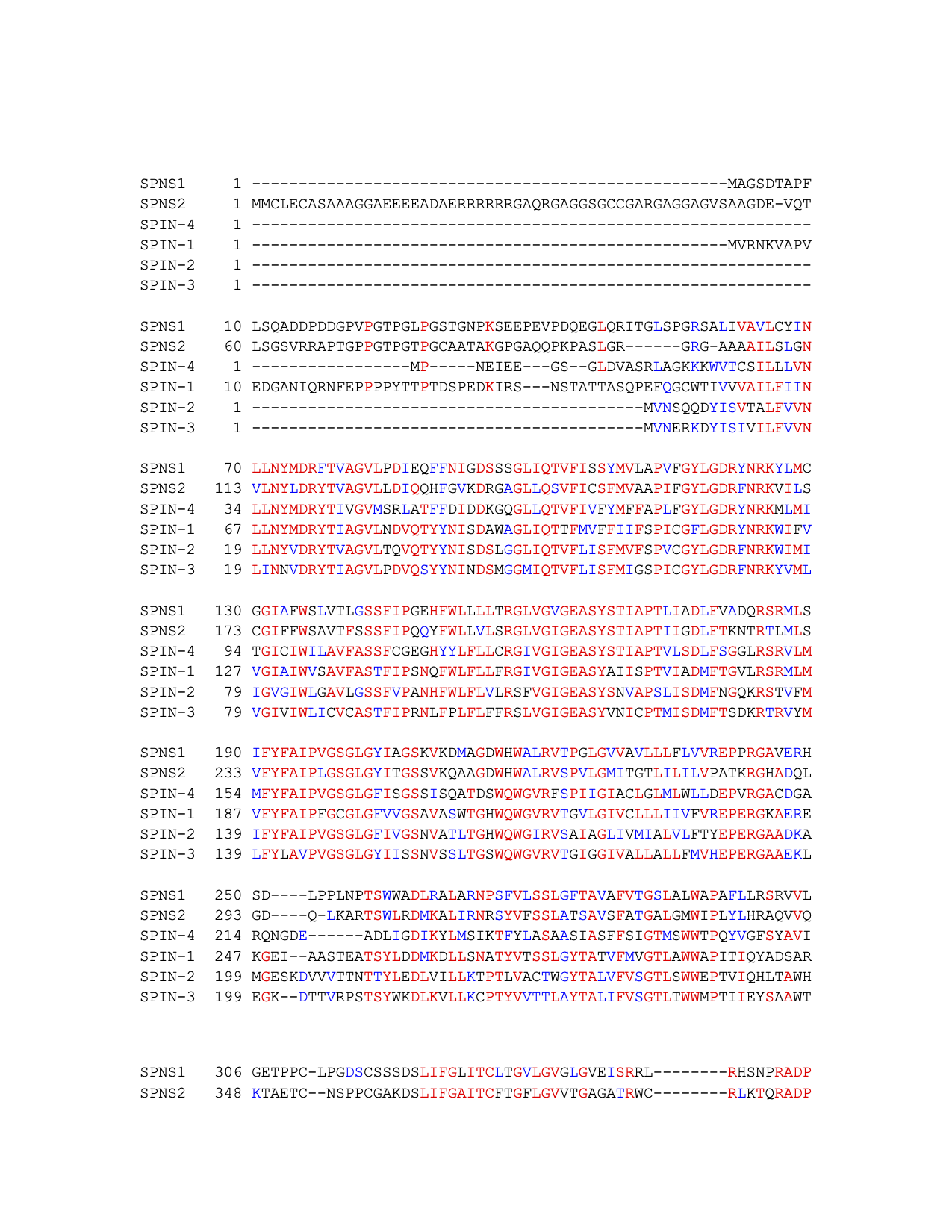

Supplement: Supplementary file 1 [file ijms-21-07813-s001.zip › Supplementary Figure 1.tiff]

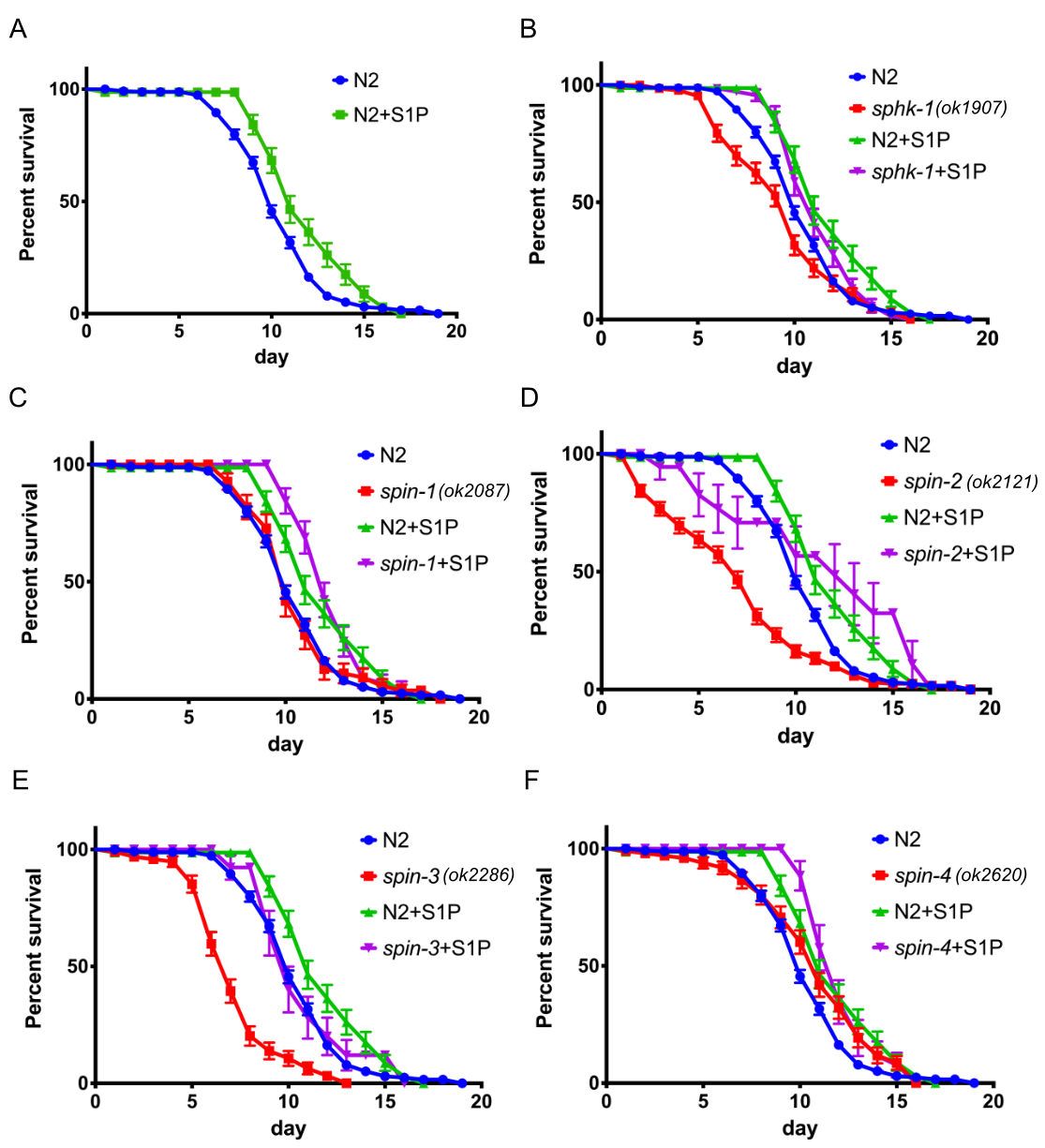

Supplement: Supplementary file 1 [file ijms-21-07813-s001.zip › Supplementary Figure 2.tiff]

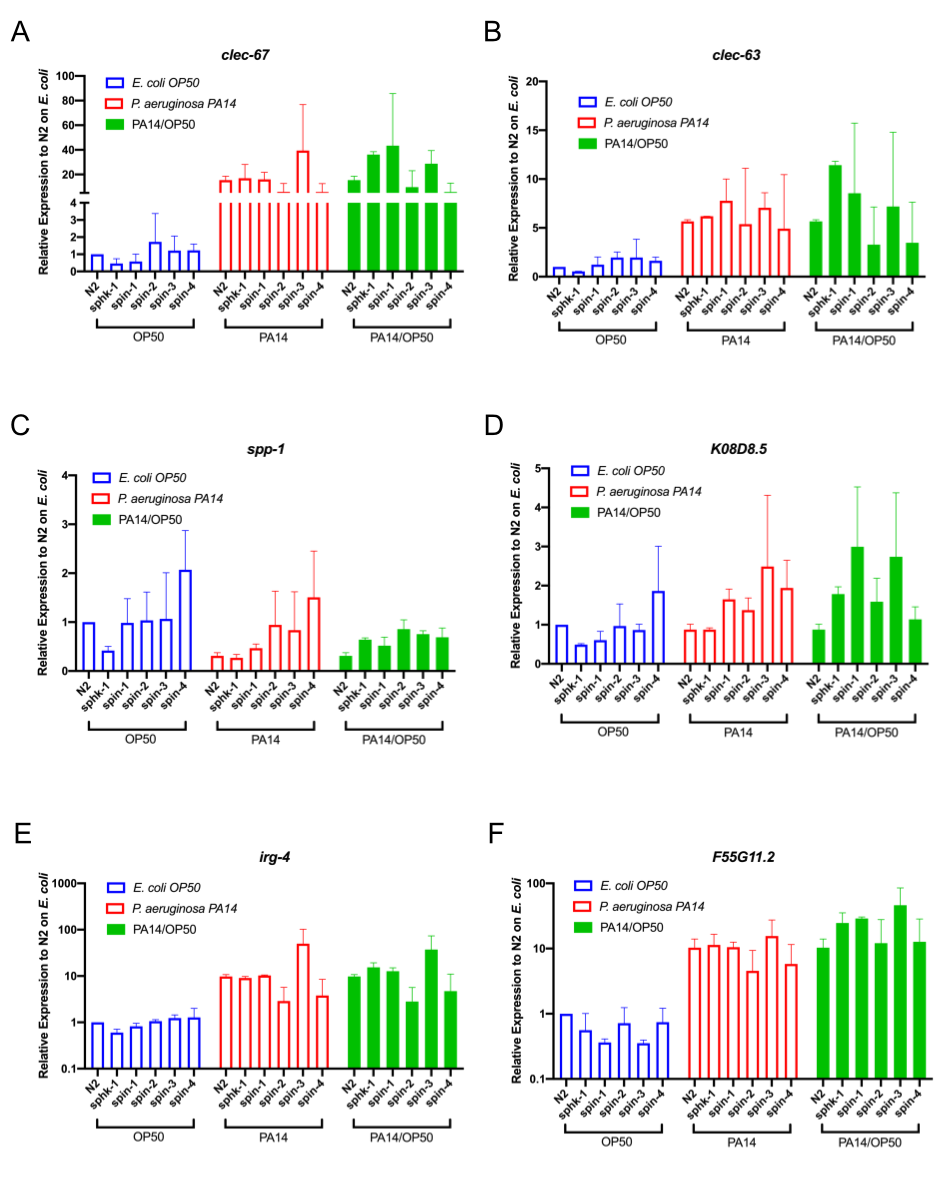

Supplement: Supplementary file 1 [file ijms-21-07813-s001.zip › Supplementary Figure 3.tiff]
